# Factors associated with lower-risk cannabis use in adults in their mid-thirties

**DOI:** 10.1186/s42238-025-00374-9

**Published:** 2025-12-10

**Authors:** Guillaume Dubé, Jennifer O’Loughlin, Jean-Sébastien Fallu, Christophe Huỳnh, Marie-Pierre Sylvestre

**Affiliations:** 1 École de santé publique de l’Université de Montréal, Montreal, Canada; 2 Département de psychoéducation de l’Université de Montréal, Montreal, Canada; 3 University Institute on Addictions (Integrated University Health and Social Services Centre of the Central-South Island of Montreal), Montreal, Canada; 4 Centre de recherche du Centre hospitalier de l’Université de Montréal, Montreal, Canada

**Keywords:** Cannabis, Lower-risk cannabis use, Epidemiology, Cannabis use disorder

## Abstract

**Background:**

Harm reduction strategies encourage people who use cannabis to adopt lower-risk behaviors, such as refraining from daily or intensive use or limiting simultaneous use with other psychoactive substances. However, little is known about the characteristics of people who use cannabis but are at lower risk of cannabis use disorder (CUD). This study identified sociodemographic, lifestyle, and mental health factors associated with lower-risk cannabis use among adults in their mid-thirties.

**Methods:**

Cross-sectional data from 731 adults in the Nicotine Dependence in Teens study were analyzed from the 2022–2023 data collection. Risk of CUD was assessed with the Cannabis Abuse Screening Test (CAST). Sociodemographic, mental health, lifestyle factors, and patterns of cannabis use were compared across participants who did not use cannabis, those with lower-risk cannabis use, and those at higher risk of CUD using cross-tabulations. Among participants who used cannabis, log-binomial regression models adjusted for age, sex, and education were fitted to identify factors associated with lower-risk cannabis use.

**Results:**

Of 731 participants (mean [SD] age = 35.3 [0.6] years; 58% female), 44% reported past-year cannabis use, including 63% classified as lower risk and 37% at higher risk of CUD. Compared with other participants, those at higher risk of CUD were more often male and had lower levels of education. Several mental health indicators were less favorable among participants at higher risk of CUD, who also reported a higher prevalence of cigarette smoking. In multivariable models, being female and simultaneous cannabis and alcohol use were associated with a higher prevalence of lower-risk cannabis use, whereas higher frequency of cannabis use, simultaneous cannabis and tobacco use, cigarette smoking, and GAD-7 scores > 10 were associated with a lower prevalence.

**Conclusion:**

Participants with lower-risk cannabis use resemble participants who do not use cannabis more than participants at higher risk of CUD. While use frequency is key, other factors, such as cigarette smoking, distinguish higher CUD risk from lower-risk cannabis use. Findings underscore the importance of harm reduction strategies and evidence-based education for cannabis-related policies.

**Supplementary Information:**

The online version contains supplementary material available at 10.1186/s42238-025-00374-9.

## Introduction

Cannabis use among Canadians age ≥ 15 years increased steadily from 5.6% in 1985 to 14.8% in 2017 (Statistics Canada 2023b). After legalization of non-medical cannabis use in 2018, it increased to 26% among Canadians age ≥ 16 years in 2023 (Statistics Canada 2023a). The 20–24 age group had the highest use in 2023, with 48% past-year prevalence (Statistics Canada 2023a). Daily or almost daily use was reported by 8.7% of adults age 18–24 and 10.3% age 25–44 in 2023 (Statistics Canada, 2024). A recent study found that 72.4% of people who use cannabis daily experienced impaired control, indicating high cannabis use disorder (CUD) risk (Rotermann 2023).

The Deficit Model, also known as the Pathology Model, is a predominant theoretical framework in investigating CUD (St-Jean et al. 2022). This model “positions illicit drug use as inherently aberrant, as destructive to both health and happiness, and as reflecting deficits in personality or social position.” (Southgate and Hopwood 1999) The Deficit Model, also applied to alcohol and nicotine, identifies risk factors for initiation and negative consequences of drug use (Barratt 2011). In cannabis use, it links to psychotic and depressive disorders, suicidal behaviour, and cognitive decline (Campeny et al. 2020; Hall 2015; World Health Organization 2016). Public policies guided by the Deficit Model advocate for prohibition and abstinence (Pacula et al. 2014).

Because 85% of people who use cannabis do not develop clinically significant dependence (Bonner et al. 2017; Connor et al. 2021; Lopez-Quintero et al. 2011), the Deficit Model is criticized for inaccurately denigrating individuals who use cannabis (Subritzky 2018). The high prevalence of cannabis use, particularly among young adults, underscores a compelling need for a paradigm shift in cannabis research (Fischer et al. 2009). Based on research indicating that public policies aimed at minimizing harm and maximizing public health could reduce problems associated with cannabis use more effectively, the Harm Reduction Model challenges the Deficit Model (Rehm and Fischer 2015), and the Lower-Risk Cannabis Use Guidelines (LRCUG) were introduced to encourage harm reduction strategies (i.e., refraining from daily or intensive use (e.g. binging; limiting simultaneous use with other psychoactive substances); avoiding or adjusting cannabis use in the presence of an active mood (e.g., depression) or substance use disorder (Fischer et al. 2011, 2022). Similar to advice on moderate alcohol consumption, nutritional practices, and sexual activity (Fischer et al. 2022), its primary aim is to provide evidence-based advice to help people who use cannabis mitigate health risks associated with cannabis use.

Despite the gradual shift towards LRCUG harm reduction, important knowledge gaps remain regarding lower-risk cannabis use. Much research has focused on problematic use and CUD, limiting understanding of characteristics associated with lower-risk cannabis use (St-Jean et al. 2022). Further there is a general lack of recognition that many people who use cannabis engage in use that does not harm their physical or mental health, social relationships, or societal participation (Subritzky 2018).

To address these gaps, our objective was to identify factors associated with lower-risk cannabis use. Specifically, we compared the characteristics of people with lower-risk cannabis use with those of people who do not use cannabis and people at higher risk of CUD, including well-established factors associated with CUD (Bastiani et al. 2017) (i.e., sex, employment status, age at onset, patterns of current cannabis consumption, simultaneous use of cannabis with other substances (Blankers et al. 2014). Consistent with earlier research (Allen et al. 2017; St-Jean et al. 2022; Subritzky 2018), increased understanding of the characteristics of people with lower-risk cannabis use can inform and enhance the effectiveness of harm reduction strategies and public health policies by raising awareness among at-risk populations and promoting healthier cannabis-related behaviours.

## Methods

### Study population

Data were drawn from the Nicotine Dependence in Teens (NDIT) Study, an ongoing longitudinal investigation initiated in 1999–2000. NDIT recruited 1,294 grade 7 students in 10 high schools located in or near Montreal, Canada. Schools were selected in non-probabilistic sampling to assure inclusion of English and French-speaking students, students living in urban, suburban or rural jurisdictions, and students with high, moderate or low socio-economic status (O’Loughlin et al. 2015). At study inception in 1999, the baseline characteristics of NDIT participants were similar to those of a provincially representative sample of youth aged 13 years from the Quebec Child and Adolescent Health and Social Survey (O’Loughlin et al. 2015). The current cross-sectional study uses data collected in online self-report questionnaires in cycle 25 conducted in 2022-23 (i.e., 4–5 years after legalization of cannabis in Canada in 2018).

NDIT was approved by the Montreal Department of Public Health Ethics Review Committee and the Ethics Research Committee of the Centre de Recherche du Centre Hospitalier de l’Université de Montréal.

### Study variable

*Current cannabis use* was measured using the prompt: “In this questionnaire, the term cannabis includes marijuana (pot, weed), hashish (hash), liquid extracts or concentrates (cannabis oil), solid extracts or concentrates (shatter, budder, wax) or any other products made from the cannabis plant, but not synthetic cannabinoids such as Spice, K2, or Yucatan Fire. Cannabis use includes smoking, vaping, eating, and consuming cannabis in any other way, whether for medical or non-medical purposes. Please think about your use of cannabis for medical or non-medical purposes. Check the one box below that describes you best.” Response choices ranged from “I have never used cannabis in my life” to “I use cannabis every day.” Participants reporting “once or a couple of times each week” or “every day” were labelled as using cannabis use frequently.

*Cannabis use a higher risk of CUD* was ascertained using the Cannabis Abuse Screening Test (CAST) which comprises six items divided into two categories: reasons to use cannabis outside of pleasure and socialization, and negative social and personal impacts associated with cannabis use (Bastiani et al. 2017). CAST items measure morning consumption, solitary consumption, memory loss, cessation attempts, perceptions of acquaintances about cannabis use, and social conflicts related to cannabis use in the past year. Response options for each question were never, rarely, from time to time, quite often, and very often, scored 0–4. Scores were summed to obtain an overall score ranging from 0 to 24, with higher scores indicating higher risk of CUD. The internal consistency of CAST in the general French population was satisfactory (Cronbach’s α = 0.74) (Legleye et al. 2015). Multiple thresholds to detect high CUD risk using the CAST have been proposed (Legleye 2018; Legleye et al. 2007, 2012, 2015). A threshold ≥ 5 optimizes a balance between sensitivity (78.2%) and specificity (79.6%) (Legleye 2018).

*Reasons for using cannabis* was assessed by: “In the past 12 months, how often did you use cannabis for …?” Response options included “medical purposes,” “recreational purposes,” “to help with symptoms of depression,” “to help with anxiety/nerves,” “to help with sleep problems,” and “to help with pain.” To help with symptoms of depression or anxiety were combined into a single category labelled “to improve mental health.” No participant reported “medical purposes,” “to help with sleep problems,” or “to help with pain” as their most frequent reason to using cannabis. Items were rated on a 5-point Likert scale from 1 (never) to 5 (every day). *Primary reason for using cannabis* was identified based on the highest frequency reported for a specific motive. “Recreational purposes” and “to improve mental health” were often reported with identical frequency. The variable was thus recoded as “recreational”, “to improve mental health” and “both recreational and to improve mental health.”

*Simultaneous use of cannabis and alcohol* or *tobacco* was measured by: “In the past 12 months, how often did you use the following substances at the same time as cannabis so that the effects overlap.” Response options were never, less than once a month, 1–3 times per month, 1–6 times per week, and every day. Due to limited use of other substances, we focused on alcohol and tobacco. If participants reported the same frequency for both current cannabis use and simultaneous use of alcohol or tobacco with cannabis, they were categorized as always using cannabis simultaneously with the specific substance (e.g., alcohol, tobacco, or both).

*Anxiety symptoms* were measured using the 7-item Generalized Anxiety Disorder Scale (GAD-7) (Spitzer et al., 2006). The total score ranges from 0 to 21, with higher scores indicating greater anxiety. Two groups were created using a validated threshold of 10 to screen for anxiety disorders (Löwe et al. 2008). The GAD-7 has a sensitivity of 0.92 and specificity of 0.70 (Johnson et al. 2019).

*Depressive symptoms* were measured with the 12-item Major Depression Inventory (MDI). The total score ranges from 0 to 50 with higher scores indicating more frequent symptoms of depression. The MDI demonstrates strong internal consistency (Cronbach’s α = 0.83) (Otiende et al. 2017). Two categories were created using a validated threshold of 21 to detect mild depressive symptoms (Bech et al. 2015).

*Emotional regulation* was assessed using the Emotion Regulation Questionnaire (ERQ), which comprises ten items (six measured cognitive reappraisal (i.e., altering one’s interpretation of a stressful situation to reduce emotional discomfort); four measuring expressive suppression (i.e., efforts to inhibit the expression of emotional discomfort) (Gross and John 2003). Higher scores on the cognitive reappraisal and expressive suppression subscales indicate more frequent use of emotion regulation strategies. Both subscales were treated as quantitative variables, with possible scores ranging from 7 to 42 for cognitive reappraisal and from 4 to 28 for expressive suppression. Both demonstrate acceptable internal consistency (Cronbach’s α and McDonald’s Ω = 0.76) (García et al. 2023).

*Coping strategies* were measured using the Coping Inventory for Stressful Situations (CISS-21) which categorizes coping styles as *avoidance*,* emotion-oriented*, or *task-oriented* (Boysan 2012). The questionnaire includes 21 items (i.e., 7 items/subscale). Participants were categorized by coping style based on the highest score among subscales (there were no ties across subscales). Cronbach’s α was > 0.72 for all subscales (Boysan 2012).

*Psychological well-being* was assessed using the Mental Health Continuum-Short Form (MHC-SF) (Keyes 2002). It includes 14 items which measure three dimensions: emotional well-being, psychological well-being, and social well-being. The scale is treated as a quantitative variable, with scores ranging from 0 to 70 with higher scores indicating greater levels of positive well-being. The MHC-SF demonstrates high internal consistency (Cronbach’s α = 0.92). (Franken et al. 2018).

*Sleep quality* was measured using the Pittsburgh Sleep Quality Index (PSQI) (Buysse et al. 1989) which assesses seven dimensions: bedtime, sleep latency, wake time, sleep duration, sleep disturbances, daytime sleepiness, and overall sleep quality. The total score ranges from 0 to 21 with higher scores indicating lower quality sleep. Participants were categorized based on a validated cutoff score of 5 to identify poor sleep quality (Mollayeva et al. 2016). The PSQI has acceptable internal consistency (Cronbach’s α = 0.70 to 0.83), test-retest reliability, and validity (Mollayeva et al. 2016).

*Moderate to vigorous physical activity* (MVPA) was measured by: “On the days that you did vigorous physical activities, how many minutes did you usually spend per day?” and “On the days that you did moderate physical activities, how many minutes did you usually spend per day?”. The responses to both questions were combined and treated as a continuous variable for analysis.

*Screen time* was measured by: “How many hours per day do you usually spend in front of a screen (computer, hand-held device) for work or for school…on weekdays?”, “How many hours per day do you usually spend in front of a screen (computer, hand-held device) for work or for school…on weekends?”, “During your leisure time, how many hours per day do you usually spend in front of a screen (computer, TV, hand-held device)…on weekdays?”, and “During your leisure time, how many hours per day do you usually spend in front of a screen (computer, TV, hand-held device)…on weekends?”. The responses to all four questions were summed and treated as a continuous variable for analysis.

### Data analysis

Descriptive statistics were used to describe the sample, identify outliers, and compare participants who do not use cannabis with participants with lower-risk cannabis use and participants at higher risk of CUD. Group differences were assessed using Chi-square (χ²) tests for categorical variables and one-way ANOVA for continuous variables. When overall tests were significant, pairwise post-hoc comparisons were conducted using Bonferroni-adjusted proportion tests or Tukey’s HSD, as appropriate.

Item-level CAST means and the proportion of participants endorsing scores ≥ 3 (“from time to time” or more often) were calculated among those who reported cannabis use, stratified by risk for CUD. To identify factors associated with lower-risk cannabis use, we estimated two models for each factor retained based on the literature - an unadjusted model and a model adjusted for age, sex, and education. Adjustments were limited to these three covariates to limit issues with model convergence and quasi-separation given that the number of participants at higher risk of CUD was relatively low (*n* = 120) (Williamson et al. 2013). This approach was also preferred over a comprehensive model that considers all factors potentially associated with lower-risk cannabis use simultaneously, to limit the risk of a Table 2 Fallacy (Westreich and Greenland 2013) in the interpretation of the estimated coefficients. Because of the cross-sectional study design, we used log-binomial models to estimate prevalence ratios. Sensitivity analyses were conducted in a subsample of participants who used cannabis more than once per week to explore whether associations with CAST scores differed in participants with more frequent cannabis use, who may be at greater risk of CUD. All analyses were conducted using R version 4.3.2 (R Core Team 2023).

## Results

### Sample characteristics

Table 1 presents the characteristics of the 731 participants retained for analysis. The mean (SD) age was 35.3 (0.6) years; 58% were female, and 94% were Canadian-born. In the past 12 months, 43.9% (*n* = 321) reported cannabis use. Among participants who used cannabis in the past year, 62.6% (*n* = 201) were classified as having lower-risk cannabis use (CAST < 5), and 37.4% (*n* = 120) were at high risk for CUD (CAST ≥ 5). Participants lost-to- follow-up were more likely to be male and higher proportions used cigarettes and alcohol at baseline compared to those not lost-to-follow-up (Supplementary Table 1).

**Table 1:** Characteristics of participants, NDIT Study, 2023, *n* = 731 participants

Variable	Proportion
Age, mean (SD)	35.3 (0.637)
Female, %	57.5
Born in Canada, %	94.1
High school diploma or less %	9.1
Household income ≤ 50,000 CAD, %	17.4
Single, %	28.4
Unemployed, %	11.7
Lives with children, %	60.7
Lives alone, %	17.8

### Comparison across cannabis use and CUD-risk groups

In terms of sociodemographic characteristics, participants at higher risk of CUD were more often male and had lower levels of education than those with lower-risk cannabis use or no cannabis use (Table 2). In addition, participants at higher risk of CUD were more likely to live alone than participants who did not use cannabis.

**Table 2:** Comparison of sociodemographic, mental health, psychological, and lifestyle characteristics across participants who do not use cannabis, participants with lower-risk cannabis use, and participants at higher risk of CUD, NDIT Study, 2023

	Participants who do not use cannabis	Participants with lower-risk cannabis use	Participants at higher risk of CUD	*P* value (χ² or Anova when continuous)
	(*N* = 410)	(*N* = 201)	(*N* = 120)	
**Sociodemographic**				
Age, mean (SD)	35.2 (0.6)	35.3 (0.7)	35.4 (0.6)	0.008 †
Female, %	62.2	57.2	41.7	< 0.001 †
High school graduate or lower, %	8.0	6.6	16.8	0.006 †
Born in Canada, %	92.4	95.5	97.5	0.071
Unemployed, %	12.1	9.6	13.8	0.505
Household income < 50,000 CAD, %	16.0	16.5	23.5	0.168
Single, %	22.6	33.9	39.7	< 0.001 ‡
Lives with children, %	67.6	51.3	50.0	< 0.001 ‡
Lives alone, %	14.8	18.0	27.6	0.007 *
**Mental health**				
Self-rated health (very good or excellent), %	77.2	79.1	75.4	0.746
Self-rated mental health (very good or excellent), %	73.3	71.9	61.9	0.053
Self-rated emotional health (very good or excellent), %	73.0	69.4	60.2	0.027 *
Positive mental health, mean (SD)	54.3 (11.4)	53.0 (11.6)	50.1 (12.7)	0.003 *
Diagnosis of mental disorders, %	26.2	34.7	35.6	0.038 §
GAD-7 ≥ 10, %	13.4	12.9	25.8	0.002 †
MDI ≥ 21, %	9.2	10.2	17.9	0.026 *
**Psychological**				
Emotion regulation				
Cognitive reappraisal, mean (SD)	28.0 (7.5)	28.0 (7.1)	27.9 (7.6)	0.97
Repression, mean (SD)	15.6 (4.7)	15.2 (4.6)	15.3 (4.9)	0.630
Coping Style				
Avoidance coping, %	48.5	54.2	44.2	0.191
Emotion-oriented coping, %	17.3	19.9	19.2	0.716
Task-oriented coping, %	34.1	25.9	36.7	0.064
**Lifestyle**				
Sleep quality (good, very good or excellent), %	59.4	63.8	55.1	0.300
PSQI > 5, %	44.1	35.3	52.5	0.009 †
Hours of moderate to vigorous physical activity per week, mean (SD)	3.2 (4.9)	4.6 (6.6)	4.9 (6.4)	0.002 ‡
Screentime hours per week, mean (SD)	13.5 (6.7)	13.3 (5.6)	15.8 (9.7)	0.006 †
Sitting hours per week, mean (SD)	11.8 (5.3)	12.0 (5.3)	12.2 (7.7)	0.738
Smoking (weekly or everyday smoker), %	3.7	12.6	36.1	< 0.001 † ‡

^† = Participants at higher risk of CUD differ significantly from both other groups in post−hoc analysis^

^‡ = Participants who do not use cannabis differ significantly from both other groups in post−hoc analysis^

^* = Participants at higher risk of CUD differ significantly from non−users only in post−hoc analysis^

^§ = Although the overall test was statistically significant, no pairwise differences reached statistical significance in post−hoc tests^

Similar proportions of participants with lower-risk cannabis use (34.7%) and participants at higher risk of CUD (35.6%) reported a diagnosed mental disorder, compared with a smaller proportion among participants who do not use cannabis (26.2%). Although the overall difference across groups was statistically significant (*p* = 0.03), post-hoc tests revealed no significant pairwise differences. GAD-7 scores **> **10 were more common in participants at higher risk of CUD. Participants at higher risk of CUD were less likely than participants who did not use cannabis to describe their emotional health as “very good” or “excellent”, and to report higher levels of positive mental health. Emotion regulation scores and coping style were similar between groups. Participants at higher risk of CUD had poorer sleep quality as measured by the PSQI, whereas self-rated sleep quality did not differ substantially across groups. Participants who did not use cannabis reported fewer minutes of moderate and vigorous physical activity (MVPA) per week than both participants at lower and higher risk of CUD. In contrast, participants at higher risk of CUD reported more hours of screen time per week compared to both lower-risk participants and participants who did not use cannabis. Compared to the other two groups, a higher proportion of participants at higher CUD risk smoked cigarettes (36.1% vs. 12.6% for participants with lower-risk cannabis use; 3.7% for participants who do not use cannabis).

To provide more detail on the components of problematic cannabis use, item-level CAST responses were examined. Participants at higher risk of CUD had higher mean scores on all six CAST items and were markedly more likely to endorse “from time to time” or more often on each item than participants with lower-risk cannabis use. Differences were especially pronounced for morning use and solitary use (e.g., 69.2% vs. 1.5% and 97.5% vs. 17.4%); see Supplementary Table 2 for descriptive statistics for all CAST items.

### Cannabis use patterns and perceived effects

Table 3 compares cannabis use characteristics in participants reporting lower-risk use with those reporting higher risk of CUD. Most participants at higher risk of CUD (90%) used cannabis weekly or daily, compared to 18% of participants with lower-risk cannabis use. Participants with lower-risk cannabis use were more likely to report using cannabis for recreational purposes and that the effect of cannabis on their mental health was neutral. Participants at higher risk of CUD were more likely than participants with lower risk cannabis use to report that using cannabis improved or worsened their mental health.

**Table 3:** Cannabis use-related characteristics by CUD-Risk status (lower-risk versus higher risk of CUD), NDIT Study, 2023

	Participants with lower-risk cannabis use(*n* = 201) %	Participants at higher risk of CUD (*n* = 120) %	*P* value (χ²)
**Frequency of cannabis use in ** **past 12 months**			< 0.0001
Once or couple of times	67.2	4.2	
Once or couple of times/month	15.4	5.8	
Once or couple of times/week	12.9	22.5	
Everyday	4.5	67.5	
**Main reason for cannabis use**			
Recreational	60.7	47.5	0.029
Improve mental health	8.0	10.8	0.504
Recreational and improve mental health	31.3	41.7	0.080
**Simultaneous use of cannabis and …**			
Alcohol, but not tobacco %	32.8	7.5	< 0.001
Tobacco, but not alcohol, %	5.5	25.0	< 0.001
Alcohol and tobacco, %	13.4	7.5	0.148
**Effect of cannabis on mental health**			< 0.001
Harmful, %	46.3	66.7	
Neutral, %	46.3	21.7	
Good, %	7.5	11.7	

### Factors associated with lower-risk cannabis use among people who used cannabis in the past year

Because adjusted models for frequency of cannabis use, recreational cannabis use, MDI, and weekly screen time did not converge, they were re-estimated without adjustment for sex.

Being female and reporting simultaneous cannabis and alcohol use were associated with a higher prevalence of lower-risk cannabis use (Table 4). In contrast, higher frequency of cannabis use, simultaneous cannabis and tobacco use, cigarette smoking, and GAD-7 scores > 10 were associated with a lower prevalence of lower-risk cannabis use.

**Table 4:** Prevalence ratios and 95% confidence intervals for the association between factors and lower-risk cannabis use among people who used cannabis, NDIT Study, 2023, (*n* = 321)

	Prevalence ratio_unadj_(95% CI)	Prevalence ratio_adj_^a^ (95% CI)
**Sociodemographic**		
Age	**0.82 [0.73**,** 0.92]**	**0.85 [0.75**,** 0.97]**
Female	**1.26 [1.06**,** 1.50]**	**1.20 [1.01**,** 1.44]**
High school graduate or lower	**0.60 [0.38**,** 0.94]**	0.69 [0.44, 1.07]
Born in Canada	1.21 [0.86, 1.69]	0.99 [0.67, 1.46]
Unemployed	0.84 [0.60, 1.17]	0.81 [0.57, 1.15]
Household income < 50,000 CAD	1.20 [0.92, 1.57]	1.20 [0.92, 1.56]
Single	1.10 [0.91, 1.33]	1.07 [0.89, 1.28]
Lives with children	1.02 [0.84, 1.23]	1.03 [0.86, 1.23]
Lives alone	0.79 [0.62, 1.02]	0.82 [0.64, 1.06]
**Cannabis use**		
**Frequency of cannabis use**		
Once or couple of times each month	**0.85 [0.72**,** 0.99]**	0.85 [0.73, 1.00]^b^
Once or couple of times each week	**0.51 [0.39**,** 0.67]**	**0.45 [0.32**,** 0.62]**^**b**^
Everyday	**0.10 [0.06**,** 0.19]**	**0.08 [0.03**,** 0.16]**^**b**^
**Main reason for cannabis use**		
Recreational	**1.23 [1.03**,** 1.46]**	1.18 [0.99, 1.40]
Improve mental health	0.87 [0.73, 1.15]	0.89 [0.64, 1.24]
Recreational and improve mental health	0.84 [69, 1.02]	0.88 [0.73, 1.06]
**Simultaneous use of cannabis and …**		
Alcohol	**1.60 [1.39**,** 1.85]**	**1.58 [1.37**,** 1.83]**^**b**^
Tobacco	**0.40 [0.24**,** 0.66]**	**0.38 [0.22**,** 0.67]**
Alcohol and tobacco	1.23 [1.00, 1.52]	**1.16 [1.07**,** 1.43]**
**Health indicators**		
Health (very good, excellent)	1.08 [0.87, 1.35]	1.09 [0.87, 1.35]
Mental health (very good, excellent)	1.20 [0.98, 1.47]	1.16 [0.95, 1.41]
Emotional health (very good, excellent)	1.17 [0.96, 1.42]	1.14 [0.95, 1.38]
Diagnosed mental disorder	0.99 [0.82, 1.18]	0.94 [0.78, 1.13]
GAD-7 ≥ 10	**0.69 [0.51**,** 0.93]**	**0.70 [0.52**,** 0.93]**
MDI ≥ 21	0.75 [0.54, 1.04]	0.76 [0.55, 1.05]
Positive mental health	1.01[1.00, 1.01]	1.00 [0.99, 1.01]
**Perceived effect of cannabis on mental health**		
Harmful	**0.74 [0.62**,** 0.87]**	**0.73 [0.61**,** 0.87**]
Neutral	**1.46 [1.25**,** 1.72]**	**1.37 [1.16**,** 1.62]**
Good	0.81 [0.57, 1.17]	1.05 [0.80, 1.38]
**Emotion regulation**		
Cognitive reappraisal	1.00 [0.99, 1.01]	1.00 [0.99, 1.01]
Repression	1.00 [0.98, 1.02]	1.00 [0.98, 1.02]
**Coping Style**		
Avoidance coping	1.16 [0.98, 1.38]	1.18 [0.99, 1.40]^b^
Emotion-oriented coping	1.02 [0.82, 1.26]	0.93 [0.76, 1.14]
Task-oriented coping	0.82 [0.67, 1.01]	0.80 [0.64, 1.01]
**Lifestyle**		
Sleep quality (good, very good, excellent)	0.87 [0.72, 1.05]	0.93 [0.78, 1.11]
PSQI > 5	**0.76 [0.63**,** 0.92]**	0.80 [0.67, 1.25]
MVPA, hr/wk	1.00 [0.98, 1.01]	1.00 [0.99, 1.02]
Screentime, hrs/wk (unit = 5 h)	0.93 [0.87, 1.00]	0.93 [0.87, 1.00]^b^
Sitting, hrs/wk	1.00 [0.98, 1.01]	1.00 [0.99, 1.01]
Smoking (daily or weekly smoker)	**0.53 [0.38**,** 0.73]**	**0.51 [0.36**,** 0.73]**

^a^Adjusted for age, sex and education

^b^Adjusted for sex and education only to enable convergence. GAD-7: General Anxiety Disorder-7, MDI: Major Depression Inventory, PSQI: Pittsburgh Sleep Quality Index, MVPA: Moderate to Vigorous Physical Activity

### Sensitivity analysis among participants who use cannabis frequently

Supplementary Table 3 presents the results of a sensitivity analysis including 143 participants who use cannabis weekly, to isolate the influence of cannabis use frequency and deepen understanding of higher risk of CUD among participants who use frequently. The results were similar to those reported in Table 4. Daily cannabis use and cigarette smoking were negatively associated with lower-risk cannabis use. In the adjusted model, avoidance coping, and simultaneous cannabis and alcohol use were positively associated with lower-risk cannabis use.

## Discussion

This study examined sociodemographic characteristics, lifestyle habits, mental health status, and cannabis-related behaviours among people in their mid-thirties. We compared those who did not use cannabis with participants who used cannabis at lower risk of CUD and those at higher risk of CUD, with the latter defined as having CAST scores greater than 5. We also identified factors associated with lower-risk cannabis use among people who had used cannabis in the past year. This is the first population-based study comparing people at higher risk of CUD and people with lower-risk cannabis use after legalization in 2018.

Aligned with research identifying frequency of use as a key indicator of higher risk of CUD (Casajuana et al. 2016), frequency of use had the strongest association with lower-risk use. The risk of progressing from lower-risk to dependent cannabis use increases with high frequency of use (Connor et al. 2021). One longitudinal study found that 17.0% of people who use cannabis weekly and 18.8% of people who use cannabis daily showed signs of dependence (Cougle et al. 2016). However, focusing solely on frequency may lead to misinterpretation of CUD risk if quantity of use is not considered (Asbridge et al. 2014). Regular use of small quantities of cannabis does not raise the likelihood of problems or impair ability to function effectively and meet societal expectations (Asbridge et al. 2014).

Differences between people at higher risk of CUD and people with lower-risk cannabis use extended beyond frequency of use. Aligned with the literature (Blankers et al. 2014; Jacobs et al. 2023; Kanyoni et al. 2015; Khan et al. 2013; Rey-Brandariz et al. 2023), females were more likely than males to abstain from cannabis or to report lower-risk use. This may relate to the higher frequencies and intensity of use among males (Cuttler et al. 2016; Khan et al. 2013). However, emerging evidence indicates that females may transition to high risk of CUD more rapidly than males (Greaves and Hemsing 2020). Neurobiological differences in the functioning of the endogenous cannabinoid systems between males and females could also underpin these differences (Greaves and Hemsing 2020).

The characteristics of NDIT participants with lower-risk cannabis use resembled those of people who do not use cannabis more closely than people at higher risk of CUD, particularly in education and income. Data on education among people who use cannabis are limited and contradictory (Millar et al. 2021). Some studies suggest that their educational attainment is similar to or higher than the general population (Copeland et al. 2001; Reilly et al. 1998; van der Pol et al. 2013b). Others report lower education levels as a risk factor for cannabis dependence (Cougle et al. 2016). One study found that individuals with at least a bachelor’s degree were more likely to be at high risk of CUD compared to those who did not complete a high school education (Fataar et al. 2023). A study of monozygotic twins, in which one was a person who use cannabis and the other was not, revealed no difference in education (Eisen et al. 2002).

Mental health symptoms among people with lower-risk cannabis use and people who do not use cannabis tended to be lower compared to people at higher risk of CUD, which aligns with findings from a population-based study (van der Pol et al. 2013). This may be because many people who frequently use cannabis do so to manage or relieve mental health symptoms (Asselin et al. 2022). Longitudinal studies have demonstrated bidirectionality of the effect, wherein mental disorders in adolescence increase the risk of cannabis use, which in turn, elevates the risk of mental disorders later in life (McGee et al. 2000; Wittchen et al. 2007). Priorities regarding the impact of cannabis on mental health is to validate its potential therapeutic effects and to reassess indicators of high risk of CUD among people using cannabis for medical purposes. (Sznitman and Room 2018).

Contradicting evidence that avoidance-coping is associated with a higher frequency of cannabis use and related problems in adolescents and college students (Blessing, et al., 2024).; Lee-Winn et al. 2018), NDIT participants who used avoidance-coping were more likely to have lower-risk cannabis use. Differences could relate to varying methods across studies. We used the CAST whereas other studies used CUDIT, which incorporates frequency of use as a criterion for high risk of CUD (Blessing, et al., 2024). Avoidance coping may lead to underestimating cannabis use impacts, suggesting misclassification in higher risk of CUD reporting.

In our sample, perceiving cannabis as harmful was associated with higher risk of CUD, whereas perceiving it as neutral was linked to a lower likelihood of high CUD risk. Among the subset of 143 participants who frequently used cannabis, the association between perceived effects and higher risk of CUD was reversed, although the 95% CIs were wide and included the null. If confirmed in larger samples, this finding could suggest that frequency of use moderates the relationship between perceived harmfulness and the risk of CUD. Alternatively, perceptions of cannabis effects may be shaped by consumption frequency. Longitudinal studies are needed to determine whether perceptions and frequency interact in shaping CUD risk.

Aligned with evidence that co-use of tobacco and cannabis is associated with a higher risk of developing CUD and psychosocial problems (Peters et al. 2012; Rabin and George 2015), people who use cannabis and who smoke were more likely to report higher risk of CUD. Evidence on this topic is limited. One experimental study reported that mixing tobacco with cannabis plant material may increase the THC inhaled per gram of cannabis (Van der Kooy et al. 2009), but this has not been confirmed in human studies. Another study suggested that simultaneous use of tobacco and cannabis could be associated with increased symptoms of cannabis dependence (Ream et al. 2008).

In our sample, participants who reported simultaneous cannabis and alcohol use were more likely to report lower-risk cannabis use. Adults who frequently use cannabis and alcohol simultaneously are more likely to do so in social settings (Boyle et al. 2023a, b). Because the CAST assesses solitary cannabis use, individuals who use both substances primarily in social contexts are likely to score lower on this item compared to those who use cannabis alone. Adults who co-use may consume smaller amounts of each substance, choose lower-potency cannabis products in co-use contexts (Gunn et al. 2022), or attribute certain problems (e.g., memory issues, concerns raised by others) to alcohol rather than cannabis. These mechanisms may help explain why our results differ from Zhu et al. (2024), who, using the CUDIT problem subscale, found that simultaneous use was positively associated with problematic cannabis use. Overall, our findings highlight the need for caution in interpreting associations between co-use and lower-risk cannabis use.

We used CAST to identify high CUD risk, although other screening tools are available (Adamson et al. 2010; Bashford et al. 2010; WHO ASSIST Working Group 2002). The lack of an official definition of high CUD risk complicates accurate identification (Casajuana et al. 2016) and questions remain about the validity of these tools. While all screening instruments assess issues related to cannabis use, attempts to reduce or stop, and morning use, CAST also considers solitary use as early signs of high CUD risk. Additionally, unlike other tools, CAST does not include frequency of use. Research is needed to establish clear definitions of high CUD risk and to replicate factors associated with lower-risk use. A comparative analysis of responses to different screening tools within the same cohort of low- and high-risk people who use cannabis could offer valuable insights.

If replicated in other studies, our study suggests that factors other than frequency of use may influence lower-risk cannabis use. Given publication of lower-risk cannabis use guidelines for the general public (e.g., avoid early initiation or synthetic cannabinoids) (Fischer et al. 2022), accurately characterizing people with lower-risk cannabis use could enhance educational interventions and the promotion of harm reduction strategies.

Limitations of this study include its cross-sectional design which limits causal inference; reliance on self-report data, which may introduce misclassification error or bias; and loss-to-follow-up, potentially leading to selection bias. Participants lost-to-follow-up were more likely to be male and use cigarettes and alcohol, which may impact the validity of the results. Additionally, the homogeneity in cohort age may limit generalizability of the findings. Some models did not converge, necessitating exclusion of sex from adjustments for confounding factors. Although we controlled for potential confounders, residual confounding may remain due to factors not measured (i.e., potency of cannabis used).

## Conclusion

In this sample of Canadian adults, most people who used cannabis engaged in lower-risk use. High frequency of use, and in particular daily use, was strongly associated with higher risk of CUD, although some people who use cannabis frequently engaged in lower-risk use. Using cannabis recreationally and in social settings could underpin this observation. Co-use of cannabis and tobacco was linked to a lower likelihood of lower-risk use, suggesting it may be an important focus for harm reduction. Our findings underscore the importance of shifting toward a harm reduction model when discussing cannabis use, especially with policymakers involved in regulating cannabis distribution. Public health campaigns should promote occasional use and communication of neutral, evidence-based information about the effects of cannabis on mental health.

## Supplementary Information

Supplementary Material 1

## Data Availability

NDIT data are available on request. Access to NDIT data is open to any university-appointed or affiliated investigator upon successful completion of the application process. Masters, doctoral and postdoctoral students may apply through their primary supervisor. To gain access, applicants must complete a data access form available on our NDIT website (www.CELPHIE.ca) and return it to the principal investigator (jennifer.oloughlin@umontreal.ca).
