# Supplementary material for: Factors associated with lower-risk cannabis use in adults in their mid-thirties
Source: J Cannabis Res. 2025 Dec 10;8:10. doi: 10.1186/s42238-025-00374-9 (PMC12802284; doi:10.1186/s42238-025-00374-9)
Supplement: Supplementary file 1 — Supplementary Material 1 [file 42238_2025_374_MOESM1_ESM.docx]

SUPPLEMENTARY MATERIAL

| **Supplementary Table 1.** Characteristics of participants at study inception (1999-2000) according to retainment status in the analytical sample, NDIT Study, 1999-2023 | | |
| --- | --- | --- |
|  | **Retained in the analytical sample** | |
|  | **Yes**  **(n=731)** | **No**  **(n=539)** |
| Female, % | 57.5 | 44.0 |
| Born in Canada, % | 94.1 | 89.1 |
| Mother attended university, %  No  Yes  NA | 48.7  40.5  10.8 | 35.1  22.3  42.7 |
| Ever smoked a cigarette, % | 25.5 | 37.6 |
| Depression score, mean (SD)^1^ | 2.1 (0.6) | 2.1 (0.6) |
| Used alcohol in past 3 months, % | 40.2 | 47.2 |
|  |  |  |
|  |  |  |

^1^Kandel DB, Davies M. Epidemiology of Depressive Mood in Adolescents: An Empirical Study. Arch Gen Psychiatry. 1982;39(10):1205–1212

| **Supplementary Table 2.** Item-level CAST means and proportion reporting ≥3^*^ (“From time to time” or more often) among participants who used cannabis in the past year, NDIT Study, 2023 (n = 321) | | | | |
| --- | --- | --- | --- | --- |
| **CAST item** | **Participants with lower-risk cannabis use** (n=201) | | **Participants at higher risk of CUD** (n=120) | |
|  | Mean | Proportion ≥3^*^ (%) | Mean | Proportion ≥3^*^ (%) |
| Morning use | 1.14 | 1.5 | 3.18 | 69.2 |
| Solitary use | 1.75 | 17.4 | 4.46 | 97.5 |
| Memory problems | 1.27 | 7.0 | 2.43 | 41.7 |
| Tried to cut down/stop | 1.00 | 0.0 | 1.69 | 20.0 |
| Others’ concern | 1.03 | 0.5 | 1.68 | 21.7 |
| Social conflicts/problems | 1.00 | 0.0 | 1.24 | 6.67 |

^*^Response options for each question were never, rarely, sometimes, quite often, and very often, scored 0-4, respectively.

| **Supplementary Table 3.** Sensitivity analysis, NDIT Study, 2023, 143 frequent cannabis users. | | |
| --- | --- | --- |
| **Potential correlates** | Prevalence ratio_unadj_ (95% CI) | Prevalence ratio_adj_^a^ (95% CI) |
| **Sociodemographic** | | |
| Age | 0.92 [0.59, 1.44] | 0.66 [0.38, 1.14] |
| Female | 1.55 [0.87, 2.76] | 1.49 [0.73, 3.03] |
| High school graduate or lower | 0.88 [0.33, 2.29] | 1.16 [0.42, 3.21] |
| Born in Canada | NA | NA |
| Unemployed, n (%) | 0.85 [0.29, 2.49] | 0.91 [0.31, 2.70] |
| Household income less than 50,000 CAD | 0.92 [0.41, 2.08] | 0.77 [0.32, 1.90] |
| Single | 1.62 [0.78, 3.36] | 2.29 [0.92, 5.69] |
| Lives with children | 1.09 [0.58, 2.06] | 1.19 [0.60, 2.36] |
| Live alone | 0.34 [0.11, 1.06] | 0.27 [0.07, 1.08] |
| **Cannabis use** | | |
| Using cannabis every day | **0.20 [0.10, 0.40]** | **0.17 [0.07, 0.39**] |
| **Reasons of cannabis use** |  |  |
| Recreational | 1.14 [0.64, 2.03] | 1.00 [0.50, 1.98] |
| Improve mental health | 0.66 [0.18, 2.43] | 0.88 [0.34, 3.23] |
| Equal use for recreational and  mental health purposes | 0.98 [0.54, 1.76] | 1.03 [0.52, 2.04] |
| **Simultaneous uses of cannabis and …** |  |  |
| Alcohol | 1.82 [0.74, 4.51] | **2.55 [1.26, 5.15]** |
| Tobacco | 0.51 [0.22, 1.22] | 0.52 [0.24, 1.39] |
| Alcohol and tobacco | 1.13 [0.41, 3.09] | 0.89 [0.23, 3.40] |
| **Health indicators** | | |
| Health (very good, excellent) | 0.80 [0.42, 1.51] | 0.86 [0.41, 1.81] |
| Mental health (very good, excellent) | 1.63 [0.80, 3.33] | 1.89 [0.82, 4.37] |
| Emotional health (very good, excellent) | 1.48 [0.75, 2.93] | 1.27 [0.62, 2.62] |
| Diagnosed mental disorder | 0.93 [0.49, 1.75] | 0.76 [0.35, 1.66] |
| GAD-7 ≥ 8 | 0.64 [0.31, 1,35] | 0.66 [0.29, 1.40] |
| MDI ≥ 21 | 0.73 [0.29, 1.88] | 0.75 [0.29, 1.52] |
| Positive mental health | 1.01 [0.99, 1.03] | 1.00 [0.97, 1.03] |
| **Perceived effect of cannabis on mental health** |  |  |
| Harmful | 1.22 [0.64, 2.33] | 1.20 [0.57, 2.54] |
| Neutral | 0.99 [0.50, 1.96] | 0.84 [0.37, 1.91] |
| Good | 0.56 [0.15, 2.08] | 0.88 [0.23, 3.35] |
| **Emotion regulation** |  |  |
| Cognitive reappraisal | 1.04 [1.00, 1.08] | 1.05 [1.00, 1.10] |
| Repression | 1.02 [0.96, 1.09] | 1.03 [0.96, 1.10] |
| **Coping Style** |  |  |
| Avoidance coping | 1.61 [0.89, 2.91] | **2.22 [1.09, 4.51]** |
| Emotion-oriented coping | 0.72 [0.31, 1.67] | 0.73 [0.30, 1.79] |
| Task-oriented coping | 0.71 [0.36, 1.39] | 0.41 [0.15, 1.13] |
| **Lifestyle** |  |  |
| Sleep quality (good, very good, excellent) | 0.53 [0.26, 1.08] | 0.70 [0.33, 1.50] |
| PSQI ≥ 5 | 0.64 [0.36, 1.15] | 0.81 [0.41, 1.60] |
| Moderate to vigorous physical activity, hr/wk | 1.01 [0.97, 1.05] | 1.00 [0.94, 1.06] |
| Screentime, hrs/wk (unit=5h) | 0.83 [0.66, 1.03] | 0.86 [0.68, 1.09] |
| Sitting, hrs/wk | 0.98 [0.93, 1.03] | 0.99 [0.94, 1.04] |
| Smoking (weekly, everyday smoker) | 0.49 [0.23, 1.04] | **0.33 [0.12, 0.89]** |
| ^a^Adjusted for age, sex and education | | |
